# Development of New Simple Compositions of Silver Inks for the Preparation of Pseudo-Reference Electrodes

**DOI:** 10.3390/bios12090761

**Published:** 2022-09-16

**Authors:** Jéssica R. Camargo, Wilson S. Fernandes-Junior, Déborah C. Azzi, Raquel G. Rocha, Lucas V. Faria, Eduardo M. Richter, Rodrigo A. A. Muñoz, Bruno C. Janegitz

**Affiliations:** 1 Department of Nature Sciences, Mathematics and Education, Federal University of São Carlos, Araras 13600-970, SP, Brazil; 2 ADB Pesquisa e Desenvolvimento, Araras 13600-140, SP, Brazil; 3 Institute of Chemistry, Federal University of Uberlândia, Uberlândia 38400-902, MG, Brazil

**Keywords:** Ag conductive ink, disposable electrodes, nail polish, shellac, onsite analysis

## Abstract

Silver materials are known to present excellent properties, such as high electrical and thermal conductivity as well as chemical stability. Silver-based inks have drawn a lot of attention for being compatible with various substrates, which can be used in the production uniform and stable pseudo-reference electrodes with low curing temperatures. Furthermore, the interest in the use of disposable electrodes has been increasing due to the low cost and the possibility of their use in point-of-care and point-of-need situations. Thus, in this work, two new inks were developed using Ag as conductive material and colorless polymers (nail polish (NP) and shellac (SL)), and applied to different substrates (screen-printed electrodes, acetate sheets, and 3D-printed electrodes) to verify the performance of the proposed inks. Measurements attained with open circuit potential (OCP) attested to the stability of the potential of the pseudo-reference proposed for 1 h. Analytical curves for β-estradiol were also obtained using the devices prepared with the proposed inks as pseudo-references electrodes, which presented satisfactory results concerning the potential stability (RSD < 2.6%). These inks are simple to prepare and present great alternatives for the development of pseudo-reference electrodes useful in the construction of disposable electrochemical systems.

## 1. Introduction

Conductive inks have received remarkable attention because of their use in the construction of flexible electronic devices, such as batteries, transistors, solar cell arrays, and electrodes [1]. Currently, printed circuits are mostly produced using metal (nano)particle-based conductive inks, which demonstrate the demand for innovative ink formulations with low cost and fast production time. For this purpose, silver-based ink has been widely used due to its advantages, such as low cost, good electrical conductivity, stability, and strong antioxidant properties [2]. As it is compatible with a range of substrates, this metal can be used in the production of uniform conductive sensors with low curing temperatures [3]. Another positive aspect is its low cost and abundance when compared to other metals, such as platinum and gold [4].

In this way, the optimal properties promote the manufacture of effective electrodes, including as a pseudo-reference/reference within three- or two-electrodes in electrochemical systems [5]. Reference electrodes play an important role in electrochemical measurements [6], for having a stable and known potential which enables the application of a controlled difference of potential between the working and reference electrodes. This is relevant in electrochemical systems because potential deviations can generate changes in the electron transfer rate of the target reaction, which can promote unwanted secondary reactions of electrolyte constituents and even the electrode material itself [6]. Therefore, the potential of the reference electrode must exhibit low variations during its lifetime (usually potential variations less than 30 mV are acceptable) [7], and silver inks are affordable alternatives to achieve this stability.

It is worth mentioning that the reference electrode used must be of simple construction, present high stability, low cost and toxicity, and be able to be used under different conditions [8,9]. In this context, the simplest reference electrode reported in the literature, which meets the requirements imposed above, is the silver-based metallic electrode [9]. Among the types of reference electrodes, some are considered to be true, which present thermodynamic equilibrium among their components. On the other hand, there are those considered to be false, also known as pseudo-reference electrodes, which do not have this internal equilibrium [10]. It is known that despite this lack of equilibrium, the potential of the pseudo-reference electrode can be surprisingly constant during experiments, being an interesting aspect to be explored in the development of simple and disposable devices [10,11]. In the literature, some proposals for making reference electrodes can be found using silver, such as those developed by Dawkins et al. and by Søpstad et al. [7,12]. Also, we can highlight devices using commercial silver inks, such as those developed by Lan et al. and Moya et al. [8,13], who studied the design of reference electrodes using paper as a substrate and inkjet printing, respectively. To develop conductive inks, a compound with conductive characteristics must be dispersed in a polymeric medium that will be responsible for the adhesion to the substrate [4,14]. Polymeric compounds, such as shellac and nail polish, have been properly employed for this purpose [14,15,16,17,18,19,20,21,22]. Nail polish [11] is a polymer that has proved to be excellent for the development of conductive inks, mainly because it is already developed to come into contact with nails without causing harm to human health. On the other hand, shellac is a natural bioadhesive polymer, which comes from a resin secreted by the female insect *Kerria lacca* and is later purified for commercial use. This polymer is also widely used in the pharmaceutical industry in the development of capsules for drugs, as it does not show toxicity in direct contact with humans. Conductive inks with shellac have been found in the literature using carbonaceous materials such as graphite and carbon black [23], and it is also known that commercial Ag inks used for making pseudo-references in general are expensive and scarce. Consequently, the development of conductive inks with Ag powder and low-cost polymers has shown to be promising.

The production of disposable and portable analytical devices is of great interest in several scenarios, such as a screening methodology in hospitals, quality control for industries, and mainly for on-site analysis [24]. Concerning reference electrodes, the use of commercial conductive inks is unfeasible for large-scale production due to their high cost. To overcome this problem, these devices can be constructed using low-cost materials [25]. Although there are some methodologies involving the use of systems with only carbon as a reference electrode, it is noted that this substrate without silver ink coating has low potential stability so that considerable variations can occur along the time [20,25,26,27]. Thus, the development of conductive silver inks for building more stable pseudo-reference electrodes is a fantastic approach to enabling large-scale production at reduced cost [7,12]. In this context, the present work reports the development of two Ag conductive inks through the dispersion of Ag powder in shellac and nail polish. To obtain the proof-of-concept of functionality in maintaining the potential between different sensors, conventional, disposable, and 3D printed devices were used.

## 2. Materials and Methods

### 2.1. Preparation of the Ag Conductive Ink

Ag inks proposed in this work were prepared using two groups of compositions with colorless nail polish (Cora^®^, Serrana, São Paulo, Brazil) and shellac (Acrilex^®^, São Bernardo do Campo, São Paulo, Brazil), and Ag powder. In this sense, eight ink compositions were created using these polymers. However, only one composition of each polymer was selected to continue the tests. To prepare the nail polish (NP) and silver ink (Ag-NP ink), 0.5 g of Ag powder (Dinâmica Química Contemporânea^®^, Indaiatuba, São Paulo, Brazil) was mixed in a double asymmetric centrifuge SpeedMixer™ Dac 150.1 FVZ-K (FlackTec Inc., Landrum, SC, USA), with 0.5 g NP in the ratio of 1:1 (*w*/*w*). Furthermore, to prepare the shellac (SL) and silver ink (named Ag-SL ink), 0.6 g of silver was mixed with 0.4 g of shellac in the ratio of 3:2 (*w*/*w*). 

### 2.2. Substrate Materials

Different electrochemical systems were used to attest the efficiency of the Ag inks developed in this work. A glassy carbon electrode (GCE), a 3D platform, and a screen-printed electrode (SPE) were used as working electrodes to perform the tests. The tests with GCE as a working electrode were performed using a glass electrochemical cell with 10 mL and a platinum counter electrode. For the construction of SPEs, the sensor used was made from the conductive ink of shellac, graphite and solvent for polyurethane of 1:1:2 (*w*/*w*), GP-SL/AC, described by Henrique et al. [20]. The SPE configuration consists of a working electrode (diameter of 4.95 mm), a counter electrode, and a pseudo-reference electrode. The analyses by SPEs were performed using aliquots of 70 µL, and to connect the SPEs system to the potentiostat, a connector designed by Orzari et al. [28] was used. For the construction of the 3D-printed working electrodes, rectangular electrodes (38 mm length × 11 mm width × 2 mm thickness) were constructed with a commercial filament composed of carbon black integrated into polylactic acid (CB/PLA) (Protopasta, WA, USA) using Flashforge Dreamer NX printer and the following parameters: layer height of 0.05 mm, 100% infill density, and a printing speed of 30 mm s^−1^ with a 0.4 mm nozzle at 220 °C and a bed temperature of 70 °C in a vertical orientation. As reference and pseudo-reference electrodes, an Ag|AgCl|KCl(sat.), a commercial Ag ink obtained from Electron Microscopy Sciences (Hatfield, PA, USA), and a system containing the developed conductive based on Ag inks were used. The measurements using 3D-printed electrodes were carried out in a 3D-printed cell developed by Cardoso et al. [29].

### 2.3. Preparation of Pseudo-Reference Electrodes Using Ag Inks

**Figure 1:**
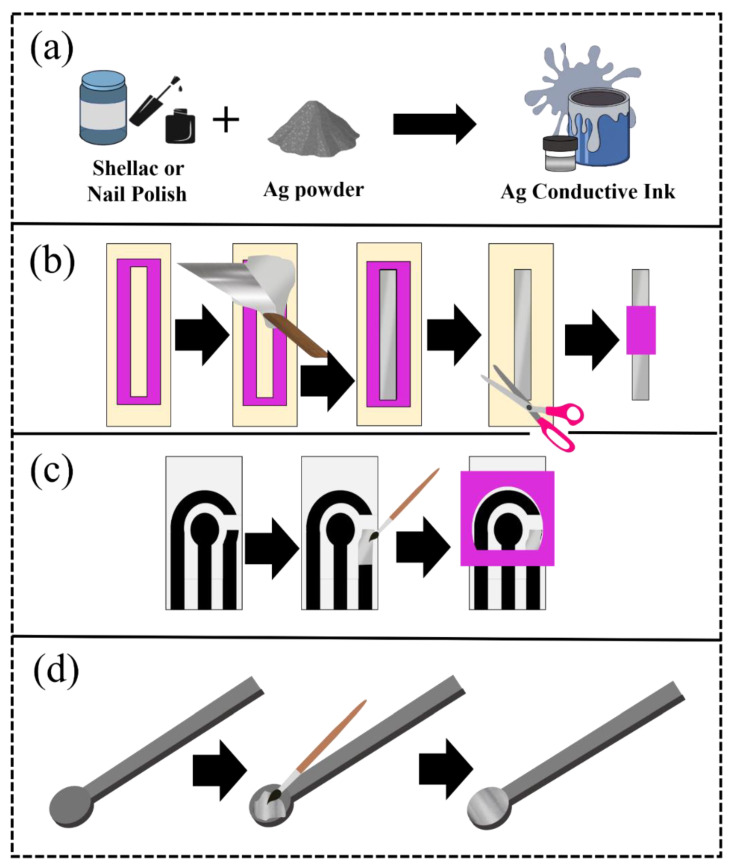
Scheme of the preparation (**a**) of the ink and the pseudo-reference electrodes, using (**b**) acetate sheets, (**c**) graphite SPE, and (**d**) 3D-printed CB/PLA as substrates.

The pseudo-reference electrodes were made with lab-made inks for using in an electrochemical cell as illustrated in Figure 1 and a video of the described production can be found in the Supplementary Material Video S1. A mask (50 mm × 6 mm), cut with a cutting printer (Silhouette, Cameo 3, Belo Horizonte, Minas Gerais, Brazil), was used on an acetate sheet to delimit the area. Then, the components were mixed using the SpeedMixer™ (FlackTek, Landrum, SC, USA), and later, this ink was spread with aid of a spatula (Figure 1a). Afterward, the mask was removed, and the excess acetate sheet was cut and a new delimiter was glued, making the contact area with the solution in the size of 20 mm × 6 mm. Thus, the reference electrodes were applied in traditional electrochemical cells (Figure 1b). SPEs systems were also developed to demonstrate the applicability of the proposed ink in different electrochemical devices. For this purpose, the ink was spread on the surface of the reference electrode with the aid of a brush (Figure 1c). The same procedure was performed using a 3D-printed CB/PLA platform, as seen in Figure 1d. 

### 2.4. Process of Electrodeposition of Silver Chloride on the Surface of the Pseudo-Reference Electrode

The silver ink deposited on the substrate was also modified with silver chloride. For this purpose, a solution of 0.1 mol L^−1^ HCl was used, and the electrodeposition was carried out, applying +1.0 V for 120 s according to the procedure described in the book by Fatibello-Filho et al. [30] for the SPEs and Ag/AS system, respectively.

### 2.5. Electrochemical Measurements

All the reagents used in this study were of analytical grade, obtained from Fisher (Hampton, VA, USA), Dinâmica (Indaiatuba, Brazil), Sigma-Aldrich (St. Luis, MO, USA), or Fluka (Buchs, Switzerland) and the solutions were prepared using purified water Heal Force^®^ (resistivity ≥ 18.2 MΩ cm). The electrochemical measurements (cyclic voltammetry (CV), open circuit potential (OCP), and square wave voltammetry (SWV)) were performed, using a potentiostat/galvanostat PGSTAT204 (Metrohm Autolab, Utrecht, The Netherlands) managed by Nova 2.1.5 software (Metrohm Autolab, Utrecht, The Netherlands, available for free download). Cyclic voltammograms were achieved using a 0.1 mmol L^−1^ ferricyanide potassium and 0.1 mol L^−1^ KCl solution (both obtained from Sigma-Aldrich^®^, St. Luis, MO, USA), as a redox probe and supporting electrolyte, respectively. 

### 2.6. Characterization

Open circuit potential (OCP) measurement was used to attest the stability of the inks on different substrates, using 0.1 mol L^−1^ KCl solution for 1 h. The experiment was carried out in a two-electrode system, in which the proposed pseudo-reference was the working electrode while Ag|AgCl|KCl(sat.) was the reference electrode.

Square wave voltammograms for the GP-SL-Ac and 3D-printed CB/PLA, referenced with the prepared inks, were obtained in 0.1 mol L^−1^ phosphate buffer saline (PBS) (pH 6.0). The parameters used in the technique were similar to those used by Pradela-Filho et al. [25] (frequency = 90 Hz, modulation amplitude = 80 mV, and step potential = 9 mV). All measurements were performed in triplicate (*n* = 3). β-estradiol stock solution 10 mmol L^−1^ was prepared in an ethanol medium. The following dilutions were made using (PBS, pH 6.0).

The morphological analysis was performed by Scanning Electron Microscopy (SEM) using a Thermo Scientific Prisma E Scanning Electron Microscope (Waltham, MA, USA) with ColorSEM Technology and integrated to energy-dispersive X-ray spectroscopy (EDS), (São Paulo, Brazil), operating at 20 kV. The X-ray diffraction (XRD) was carried out by a Miniflex II X-ray diffractometer (Rigaku), from 20° to 80° angular recording range. Finally, using the equipment developed by Silva et al. [31], the contact angle analysis was accomplished. Electrochemical Impedance spectroscopy (EIS) was carried out using the 3D acetate sheets and SPE-type painted with the developed Ag inks as the working electrode, platinum as counter, and Ag|AgCl|KCl(sat.) was the reference electrode. A solution of 1.0 mmol L^−1^ [Fe(CN_6_)]^3−/4−^ and 0.1 mol L^−1^ KCl, as redox probe and supporting electrolyte and the potential applied was 0.1 V. In addition, spectra were obtained by Fourier Transform Infrared Spectroscopy (FTIR) with tensor II spectrophotometer (Bruker), with the resolution of 4.0 cm^−1^ using absorbance mode in the range of 400 to 4000 cm^−1^.

## 3. Results and Discussion

### 3.1. Silver Ink Compositions

**Figure 2:**
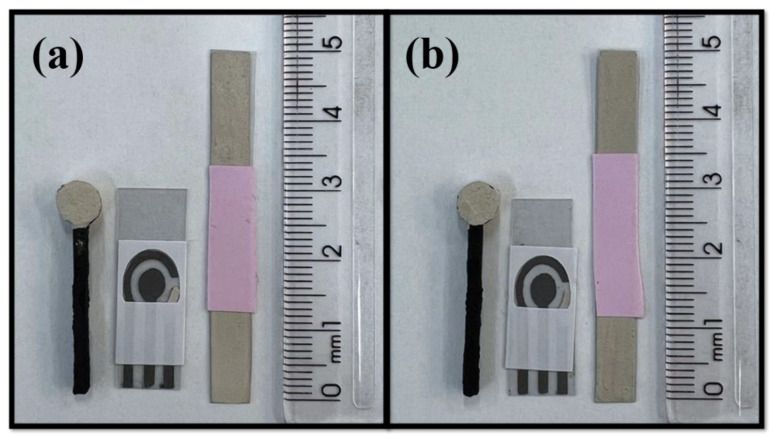
Pictures of the produced pseudo-reference electrodes with 3D-printed CB/PLA electrode, graphite SPE, and acetate sheets using (**a**) Ag-NP and (**b**) Ag-SL inks.

For the formulation of conductive inks, it is necessary to evaluate the proportions between the polymeric vehicle and the conductive material [20,32]. If necessary, other additives can be added, mainly to control viscosity and drying time [33]. In this sense, eight compositions were tested using two different types of polymers (i.e., shellac and colorless nail polish). The optimization proportions can be seen in Supplementary Material (Table S1). Inks 1, 2, and 3 were made with NP and Ag powder. Evaluating the inks by physical aspect, the best composition obtained with the NP was ink 1, with Ag powder and NP in the ratio of 1:1 (*w*/*w*), which was homogeneous in the spreading tests, while formulations 2 and 3 (ratios 3:2 and 3:7 (*w*/*w*), respectively) were not homogeneous and some regions did not present silver during the spreading tests. It is important to mention that the compositions made from NP required additives since NP has solvents in its composition and these are sufficient to control the drying time and to provide the necessary adhesion for the ink. On the other hand, the addition of solvents can be interesting to improve drying time and ink viscosity when using SL as polymer, as reported in the literature [20]. Therefore, the use of solvents for SL was tested. Inks 4, 5, 6, 7, and 8 were made with shellac (SL) Acrilex^®^. Inks 4 and 5 contained 33 and 50% of solvent (*w*/*w*), respectively, and presented a homogeneous appearance. However, their use was unfeasible for application in disposable sensors, since the solvent, before drying, also diluted the conductive graphite ink of the system. Therefore, in the case of inks 6, 7, and 8, only the mixture of SL and Ag powder was tested. SL is a more fluid polymeric base than NP, and the proportion of ink 6 had a small amount of Ag, making it difficult for the sensors to be homogeneous. On the other hand, ink 7 was very thick and made it difficult to spread, so ink 8 was chosen, since the reference electrodes obtained were homogeneous and filled with silver, in addition to not interfering with the graphite system used to manufacture the other two electrodes (counter and working electrodes). Thus, inks 1, named Ag-NP ink, and 8, named Ag-SL ink, were selected to continue the work. Figure 2 shows pictures of the systems modified with the conductive Ag inks.

### 3.2. Morphological Characterization of the Ag Conductive Inks

**Figure 3:**
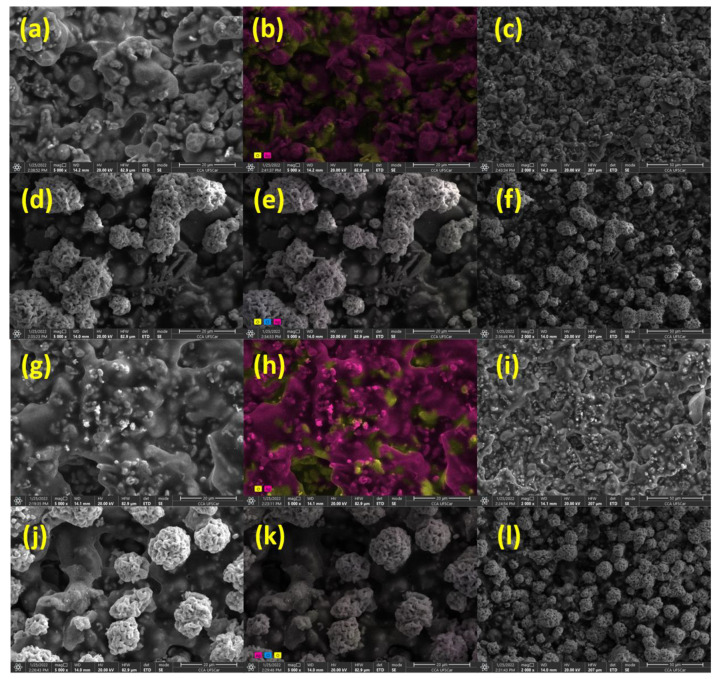
(**a**) SEM images obtained for Ag-NP ink at 5000× magnification; (**b**) ColorSEM mapping of Ag-NP ink 5000× magnification; (**c**) and Ag-NP ink at 2000× magnification; (**d**) Ag-NP ink with AgCl deposited at 5000× magnification; (**e**) ColorSEM mapping of Ag-NP ink with AgCl deposited at magnification 5000×; (**f**) and Ag-NP ink with AgCl deposited at 2000× magnification; (**g**) Ag-SL ink at 5000× magnification; (**h**) ColorSEM mapping of Ag-SL ink (magnification 5000×); (**i**) and Ag-SL ink in 2000×; (**j**) Ag-SL ink with AgCl deposited at 5000× magnification; (**k**) ColorSEM mapping of the Ag-SL ink with AgCl deposited at 5000× magnification; (**l**) and Ag-SL ink with AgCl deposited at 2000× magnification.

For morphological analysis of these inks (Ag-NP and Ag-SL), SEM measurements were performed. The SEM images showed a homogeneous and rough surface for Ag-NP ink, as observed in Figure 3a,c and the ColorSEM mapping (Figure 3b) displayed the predominance of Ag and oxygen on the electrode surface. This is likely due to the oxygen from the air playing a significant role in the dispersion of the Ag particles. According to Wiley and co-workers [34], oxidative etching helps to promote the initial dissolution and dispersion of Ag particles in the polymeric matrix.

In addition, after the deposition of AgCl on Ag-NP inks, it was possible to observe a rough surface and some AgCl agglomerates on the surface (Figure 3d). The results match with the ColorSEM mapping that was performed (Figure 3e), which shows the predominance of chlorine, silver, and oxygen on the electrode surface after the deposition.

Similar to Ag-NP ink, SEM images were obtained for Ag-SL ink and a homogeneous and rough surface was also presented (Figure 3g,i). The ColorSEM mapping (Figure 3h) displayed the predominance of silver and oxygen on the electrode surface as also observed in the literature [34]. In addition, the SEM images obtained after the deposition of AgCl revealed a rough surface, with spherical AgCl clusters. The ColorSEM mapping that was performed (Figure 3k) showed the predominance of chlorine, silver, and oxygen on the electrode surface.

The EDS measurements are in accordance with the results obtained through color mappings (Figure 3) and displayed the predominance of Ag and oxygen elements on the electrode surface (Figure S1a,c). After the electrodeposition of silver chloride, the EDS also confirmed the deposition of AgCl on the surface of the pseudo-reference (Figure S1b,d). 

**Figure 4:**
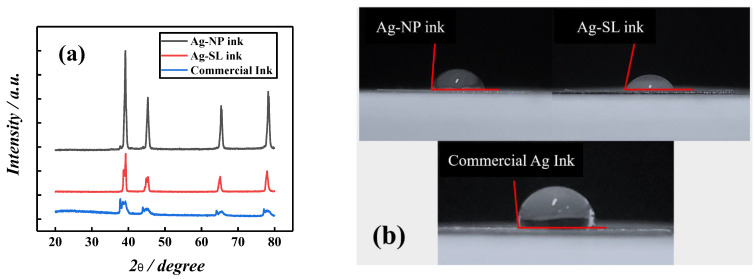
(**a**) X-ray spectrum of conductive silver inks. (**b**) The contact angle for Ag-NP (86°), Ag-SL (79°), and commercial (99°) inks spread on acetate sheets.

Additionally, X-ray diffraction measurements of the proposed and commercial inks were carried out, as shown in Figure 4a. The diffraction patterns show four sharp and well-defined lines of diffraction at 2θ = 39.25°, 44.22°, 65.33°, and 78.23°, which can be attributed to the reflections of the face-centered cubic structure (fcc), (111), (200), (220), and (311) of metallic silver for all inks. These results are similar to those obtained in the literature for silver particles [35,36,37] and demonstrate that these silver particles are available, and the polymers used for the composition of inks do not interfere with their functionality. The contact angle measurement was performed, using the system developed by Silva et al. [31]. This study allows verifying the hydrophobic or hydrophilic character of the surface. To perform this experiment, the inks were spread on acetate sheets the size of 1.0 cm^2^. To measure the contact angles, an aliquot of 50 µL of ultrapure water was dripped onto the surface of commercial ink, Ag-NP ink, and Ag-SL ink, respectively. The photos were taken the 30s after the cast. Depending on the verified contact angle (θ) with the water, the surface can be classified as super-hydrophilic (θ < 10°), hydrophilic (θ < 90°), hydrophobic (90° < θ < 150°), or superhydrophobic (θ > 150°). Thus, the surfaces with all the inks presented hydrophobic profiles, with contact angles of 86 ± 1°, 79 ± 1°, and 99 ± 1°, for Ag-NP, Ag-SL, and commercial ink, respectively (Figure 4b). 

The EDS measurements, which can be found in Figure S1 of the Supplementary Material, show the different percentages of elemental composition on the surface of the tested inks. In this way, we consider that the different contact angles may be due to the percentage of Ag and oxygen in the inks developed in this work, or fluorine in the commercial ink [38]. 

The Adhesion Tape Tests were performed with the SPE system with the Ag developed inks. Inspired by the ASTM Ink Adhesion Tape Test technique [39,40], an adhesive tape was glued and removed over the SPE-type sensors, modified with the Ag inks developed. The adhesive tape used consists of a bioriented polypropylene film and synthetic rubber. The adhesion of the adhesive was performed by pulling it off rapidly (not jerked) in a transverse direction (45°) and vertically (90°). In Figure S2a is represented the image of the adhesive removed at 45°, and Figure S2b represents removal at 90° using the carbonaceous ink of graphite and SL and the silver ink Ag-SL. Figure S2c,d shows the photos obtained from the adhesive removed at 45° and 90° using the carbonaceous ink of graphite and SL and the silver ink Ag-NP. It can be seen that, compared to the carbon ink used to make the SPE-type devices, both developed silver inks remained adhered to the substrate, obtained only trace pelling.

**Figure 5:**
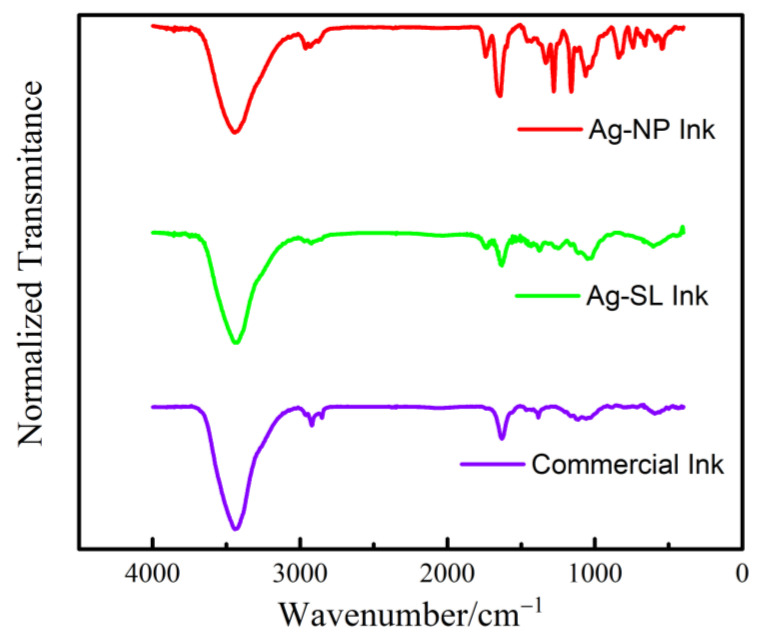
FTIR spectrum obtained for Ag-NP ink, Ag-SL ink, and Commercial Ink.

Spectrum were obtained by Fourier Transform Infrared Spectroscopy (FTIR) with tensor II spectrophotometer (Bruker), with the resolution of 4.0 cm^−1^ using absorbance mode in the range of 400 to 4000 cm^−1^. The differences among the bands found in the Ag-SL and Ag-NP inks developed and the commercial one is justified due to the different polymers and solvents implemented in each case, see Figure 5. It is possible to observe common bands among the three spectra, such as O-H bands at 3430 cm^−1^, attributed to hydrogen bonds, possibly due to air humidity [3,4]. The spectra observed for Ag-SL ink can be assigned by the bands of C=O stretching of ester 1744 cm^−1^ and O-H stretching of the hydroxyl group at 3433 cm^−1^. The intensity of the band at 2930 cm^−1^ can be related to the C-H stretching vibration [5]. Some bands, from 500 to 1800 cm^−1^, confirm the presence of ketones, alcohols, and carboxylic acids in the polymer structure. The spectra observed for Ag-NP ink C-H bands at 2965 cm^−1^, referring to alkanes (stretch); C=C bands at 1646 cm^−1^, related to alkenes (stretch); CH3 bands (bend) at 1328 cm^−1^; and C-O (stretch) bands at 1160 cm^−1^, referring to saturated alcohols. The commercial ink spectra present bands of 2930 cm^−1^ that can be due to the C-H stretching vibration; C=C bands at 1629 cm^−1^, can be related to alkenes (stretch); and C-C can be related to the 1382 cm^−1^.

**Figure 6:**
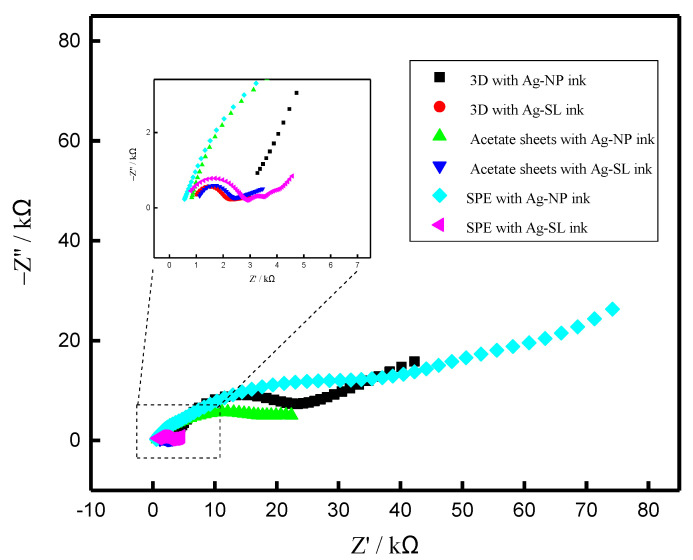
Nyquist diagrams for (black) 3D with Ag-NP ink; (red) 3D with Ag-SL ink; (green) acetate sheets with Ag-NP ink; (blue) Acetate sheets with Ag-SL ink; (cyan) SPE with Ag-NP ink and (pink) SPE with Ag-SL ink. The experiments were carried out using a solution of 1.0 mmol L^−1^ [Fe(CN_6_)]^3−/4−^ and 0.1 mol L^−1^ KCl, as redox probe and supporting electrolyte and E = 0.1 V.

Electrochemical Impedance spectroscopy (EIS), (PGSTAT204, with FRA32M, Metrohm Autolab, Utrecht, The Netherlands) was also employed to evaluate the electrical resistance of each ink, and the Nyquist plots are presented in Figure 6. According to the EIS experiments, it is possible to observe that the ink made with NP presented a surface with higher resistance to electric current in relation to the composition developed from SL for all systems tested (3D, acetate sheets, and SPE). On the other hand, it is not possible to compare the resistance between the different electrodes (SPE, acetate, and 3D) because they present different areas. The resistance values obtained are presented in Table S2, which also presents the chi-square values. The equivalent circuits obtained are shown in Figure S3. 

### 3.3. Application of Silver Inks as Pseudo-References

**Figure 7:**
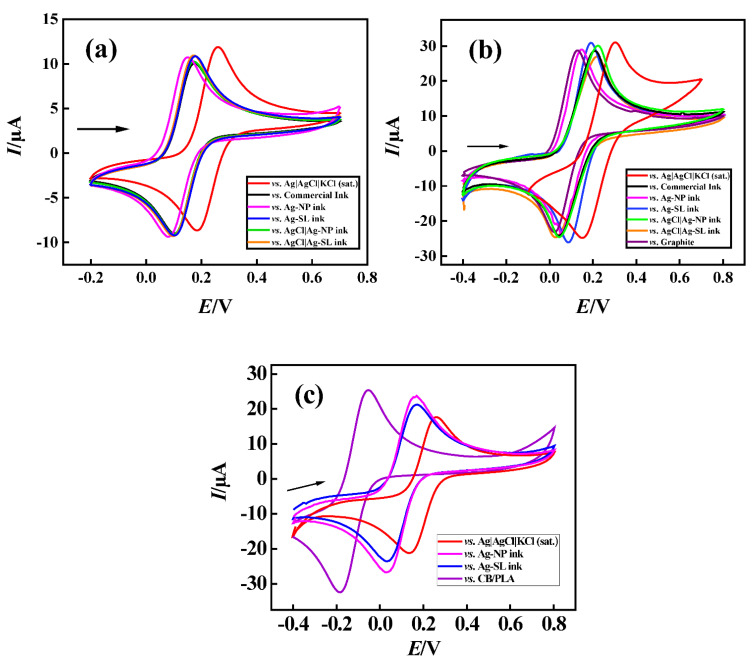
The cyclic voltametric response obtained for 1.0 mmol L^−1^ [Fe(CN)_6_]^3−/4−^varying reference electrode. (**a**) GCE vs. (red line) Ag|AgCl|KCl(sat.); (black) commercial ink; (pink) Ag-NP ink; (blue) Ag-SL ink; (green) AgCl|Ag-NP ink; and (orange) AgCl|Ag-SL ink. (**b**) GP-SL/AC vs. (red line) Ag|AgCl|KCl(sat.); (black) commercial ink; (pink) Ag-NP ink; (blue) Ag-SL ink; (green) AgCl|Ag-NP ink; (orange) AgCl|Ag-SL ink; and (purple) graphite ink. (**c**) 3D-printed CB/PLA vs. (red line) Ag|AgCl|KCl(sat.); (black) commercial ink; (pink) Ag-NP ink; (blue) Ag-SL ink; (purple) CB/PLA; using 0.1 mol L^−1^ KCl as supporting electrolyte scan rate: 50 mV s^−1^.

To demonstrate the functionality of the pseudo-reference electrodes prepared with the proposed Ag ink, cyclic voltammograms, using 1.0 mmol L^−1^ [Fe(CN_6_)]^3−/4−^ and 0.1 mol L^−1^ KCl as redox probe and supporting electrolyte, respectively, were recorded (Figure 7). Firstly, GCE and platinum were used as working and auxiliary electrodes (Figure 7a). Pseudo-reference electrodes with commercial ink were produced similarly as described in Section 2.2. 

It could be observed that both developed inks maintained the potential when compared to the commercial reference electrode, (around +0.1 V for reduction and +0.17 V for oxidation processes of [Fe(CN)_6_]^3−/4−^) with peaks shifted by 100 mV due to the difference of potential between the Ag inks and conventional reference electrodes. Moreover, peak-to-peak separation (ΔE) was ~70 mV. This difference can be explained mainly by the high concentration of chloride present in the solution, and consequently chloride species surrounding the electrode surface and promoting an equilibrium between the chloride in the solution and the silver chloride deposited on the surface [41].

Subsequently, the pseudo-references were tested in disposable electrodes made of shellac and graphite [20] (Figure 7b). Both silver inks that were developed, maintained the potential at around +0.05 V and +0.18 V for reduction and oxidation processes, respectively, resulting in a ΔE of 100 to 190 mV on average for the redox probe [Fe(CN)_6_]^3−/4−^. Much like the previous study, a potential discrepancy of 100 mV on average was achieved when compared to the conventional reference electrode Ag|AgCl|KCl(sat.). The carbon electrode was also evaluated as a reference, and it can be seen that it did not show a significant difference. However, as already justified, carbon does not keep the potential stable for a long time, making it necessary to replace the system frequently. The developed pseudo-reference electrodes showed potential values close to each other and it is considered that they were efficient to keeping the potential stable.

Finally, a third system was investigated using the 3D-printed CB/PLA platform with two Ag inks (Ag-NP and Ag-SL). As well as in previous systems, the potential was maintained close to +0.05 V for reduction and +0.17 V for the oxidation processes of [Fe(CN)_6_]^3−/4−^. Moreover, the performance of the Ag inks on CB/PLA platforms was similar to conventional Ag|AgCl|KCl(sat.), with peaks shifted by ~100 mV and ∆E was 120 mV for both electrodes.

A 3D-printed CB/PLA substrate without the conductive inks was also tested as a reference, and it can be seen that it did show a significant difference (i.e., 325 and 220 mV) when compared with conventional Ag|AgCl|KCl(sat.) and the developed devices using conductive inks, respectively, demonstrating the functionality of the conductive Ag inks in these devices.

### 3.4. Stability Assessment

**Figure 8:**
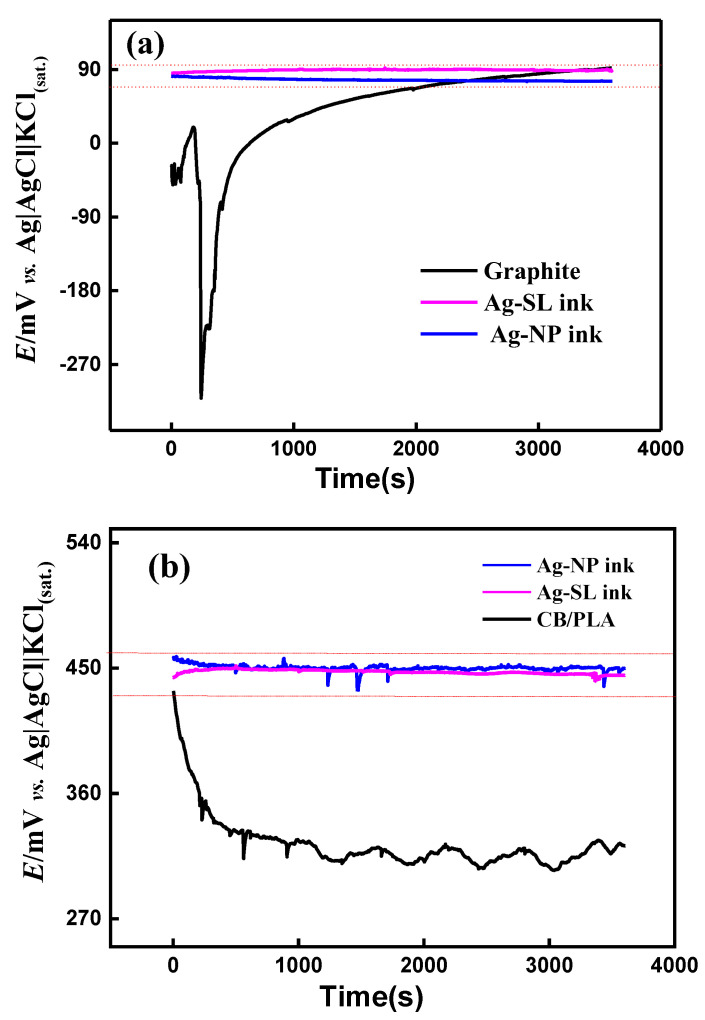
OCP measurements recorded for (**a**) GP-SL/AC system using graphite-SL, Ag-SL ink, and Ag-NP ink as working electrodes and (**b**) 3D-printed CB/PLA system, using CB/PLA, Ag-SL ink, and Ag-NP ink as the working electrode. In both systems Ag|AgCl|KCl(sat.) was used as the reference electrode and 0.1 mol L^−1^ KCl solution as the supporting electrolyte.

The stability of the potential of the pseudo-reference electrodes was evaluated in the interval of 1 h by the OCP technique. This methodology was similar to those demonstrated in the articles by Dawkins et al. and by Søpstad et al. [7,12]. A two-electrode system was used as follows: Ag|AgCl|KCl(sat.) as the reference, and the devices coated with the proposed Ag inks (Figure 1c,d) as the working electrode. Thus, an OCP was performed (Figure 8a,b) and it can be seen that in the systems in which the developed silver inks were used, the potential was kept close to 90 mV for the SPE and 450 mV for 3D-printed electrodes. 

According to the literature, a variation greater than 30 mV indicates that the electrode is not suitable for using as a reference one [42]. The pseudo-reference electrodes produced on different substrates using the proposed silver inks provided relatively constant potentials (variations less than 30 mV) throughout the evaluated period (~1 h). Thus, the fabricated silver inks can be considered a powerful tool for this purpose. The substrates without the conductive Ag inks (only carbon) exhibited considerable potential variations (~400 mV for GP-SL/AC system and ~130 mV for 3D-printed CB/PLA system) during the measurements, confirming the effectiveness of the silver inks. In addition, to confirm the long-term stability of the pseudo-reference electrodes, successive measurements (*n* = 100) with CV using 1.0 mmol L^−1^ [Fe(CN_6_)]^3−/4−^and 0.1 mol L^−1^ KCl as redox probe and supporting electrolyte were carried out (Figure S4). Potential deviations smaller than 28 mV for all pseudo-reference electrodes were achieved, which attested to their stability in the presence of redox processes. Stabilization measurements were also performed over 5 h for the SPE system, and results with variation below 30 mV were maintained, as can be seen in Figure S5.

### 3.5. Proof of Concept of the Application of Silver Inks as Pseudo-Reference

β-estradiol is an essential hormone for the human body, mainly for the development and maintenance of reproductive tissue, as well as significant effects on the brain and bones [43]. In addition to the beneficial role of β -estradiol, it is also considered an endocrine disruptor. Thus, accurate detection of β-estradiol is necessary [44]. Therefore, we seek to develop architectures that allow rapid diagnosis and portability, which directly help in the quality of life.

**Figure 9:**
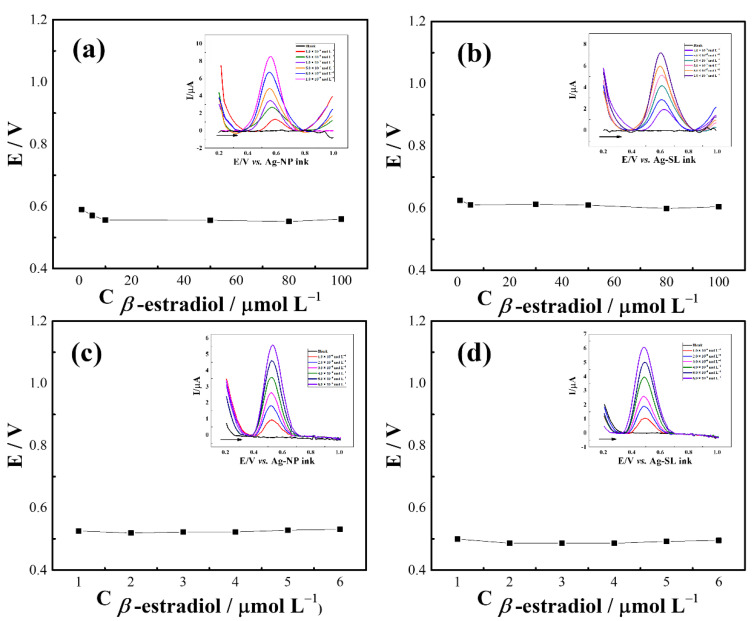
Potential (V) vs. concentration plot of the developed silver inks for GP-SL/Ac system referenced by (**a**) Ag-NP and (**b**) Ag-SL inks; and 3D-printed CB/PLA system referenced by (**c**) Ag-NP and (**d**) Ag-SL inks; insets: SWV recorded at different β-estradiol concentrations in 0.1 mol L^−1^ PBS (pH 6.0). SWV conditions: frequency = 90 Hz, modulation amplitude = 80 mV, and step potential = 9 mV.

To evaluate the applicability of the developed pseudo-references, SWV analysis were performed by constructing calibration plots using β-estradiol as a model molecule. For GP-SL/AC system referenced by Ag-NP (Figure 9a) and Ag-SL (Figure 9b) inks, good linearity (R^2^ > 0.992) in the range of 1 to 100 µmol L^−1^ and suitable potential stability (RSD < 2.6%) were achieved. The respective linear regression equations were *I* (µA) = 0.07 C (β-estradiol) + 1.56 × 10^−6^ (Figure S6a) and *I* (µA) = 0.04 C (β-estradiol) + 2.70 × 10^−6^ (Figure S6b). Using the 3D-printed CB/PLA electrode, linear ranges of 1.0 to 6.0 mmol L^−1^(R^2^ > 0.998) were obtained when the system was referenced by Ag-NP (Figure 9c) and Ag-SL inks (Figure 9d). As observed above, the potential remained practically constant (RSD < 1.2%) even after successive additions of concentrations of β-estradiol, indicating the reliability and usefulness of the proposed inks for making disposable pseudo-reference electrodes for analytical applications. The linear regression equations were *I* (µA) = 0.87 C (β-estradiol) + 6.27 × 10^−8^ and *I* (µA) = 1.01 C (β-estradiol) − 9.23 × 10^−8^, with R^2^ = 0.999 the for analysis referenced by Ag-NP (Figure S7a) and Ag-SL (Figure S7b) inks, respectively. 

The characteristics of the proposed inks were compared with other Ag-based conductive inks found in the literature, as shown in Table 1. It can be noticed that most of the developed inks use nanoparticulate silver and, in general, this process involves the synthesis of nanoparticles, in addition to a suitable time and temperature for sintering. Therefore, it is considered that the Ag conductive inks developed in this work can be applied to facilitate the production of pseudo-reference electrodes, mainly because they do not require syntheses in their process, nor do they require toxic additives and solvents or high temperatures for curing and sintering. Furthermore, as it is simple to synthesize, the developed inks also proved to be economically viable, costing about USD 2.02 per gram for nail polish and USD 2.40 for shellac.

## 4. Conclusions

Two metallic silver conductive inks were prepared and applied to three different systems, including a traditional electrochemical cell, a 3D-printed system, and a disposable system. Both produced ink compositions using nail polish and shellac, and were successfully applied to the development of pseudo-reference electrodes. In addition to being easy to manufacture and using, these inks enable the creation of more robust systems, which, even in disposable systems, can be used for a longer time without interfering with the potential. In this way, through electrochemical characterizations it was verified using OCP and cyclic voltammetry that the potential was maintained even after one hour. In addition, the SEM measurements showed that the electrodeposition of AgCl on the surface was efficient and that the prepared inks are homogeneous. In the X-ray analysis, it was possible to verify a diffraction pattern similar to commercial silver ink. For these reasons, it is believed that these new inks can be sold at a lower cost than the current ones on the market, in addition to being able to be applied in various types of sensory systems.

## Figures and Tables

**Table 1:** Comparison between the silver inks proposed for the manufacture of pseudo-reference electrodes.

Ink	Ag Particle Size	Polymer	Additives	Curing Temperature	Conductivity (Ω·m)	Ref.
Ag/AgCl ink	Micro	cellulose acetate	Cyclohexanone, acetone	90 °C	1.7 × 10^−4^	[45]
I-6	Nano	-	Ethylene glycol, water, ethanol, ethanolamine, hyperdispersant	Room	1.6 × 10^−6^	[46]
AgNP ink	Nano	PVP	Ethylene glycol/Methanol	Room	1.6 × 10^−2^	[47]
Ag-NP ink	Micro	Colorless nail polish	-	Room	2.3 × 10^−3^	This work
Ag-SL ink	Micro	Shellac	-	Room	4.5 × 10^−3^	This work

Ag/AgCl ink: nanoparticles made from AgNO_3_; cellulose acetate, acetone, and cyclohexanone as solvents; I-6: Silver nanoparticles with ethylene glycol, water, ethanol, ethanolamine, hyperdispersant (Solsperse 20,000); AgNP ink: silver nanoparticles with ethylene glycol/methanol and PVP (polyvinylpyrrolidone).

## Data Availability

Not applicable.

## Supplementary Materials

The following supporting information can be downloaded at: https://www.mdpi.com/article/10.3390/bios12090761/s1, Table S1: Compositions for the production of conductive silver ink; Table S2: Charge transfer resistance values for proposed Ag inks; Figure S1: EDS mapping of (a) Ag-NP ink; (b) Ag-NP ink with AgCl deposited; (c) Ag-SL ink; (d) Ag-SL ink with AgCl deposited and (e) Ag Commercial Ink; Figure S2: Pictures of Adhesive Tape Test were performed using the SPE-type system and the conductive Ag inks used over the reference electrode. (a) represented the image of the adhesive removed at 45°, and (b) 90° using the carbonaceous ink of Graphite and SL and the Ag-SL ink. (c,d) represented the image obtained from the adhesive removed at 45° and 90°, respectively, using the carbonaceous ink of Graphite and SL and Ag-NP ink. Inset: photos of SPE devices after pulling; Figure S3: Equivalent circuits obtained after EIS technique for the Ag inks with systems (3D, acetate sheets and SPE); Figure S4: Successive cyclic voltametric measurements (*n* = 100) obtained for 1.0 mmol L^−1^ ferrocenoethanol—varying reference electrode. (a,b) Working electrode: GP-SL/Ac; (c,d) 3D-printed CB/PLA; Scan rate: 100 mV s; Figure S5: OCP measurements recorded for 5 h in the GP-SL/AC system using Ag-NP ink and Ag-SL ink as working electrodes, Ag|AgCl|KCl(sat.) as the reference electrode and 0.1 mol L^−1^ KCl solution as the supporting electrolyte; Figure S6: Square wave voltammograms in the presence of β-estradiol, (a) using Ag-NP ink as a reference, (b) Ag-SL ink as a reference, at concentrations from 1.0 to 100 μmol L^−1^ in PBS 0.1 mol L^−1^ (pH 6.0); Step = 9 mV s^−1^; amplitude = 80 mV s^−1^; frequency = 90 mV s^−1^; (b) Plot of analytical curve for β-estradiol; Figure S7: Square wave voltammograms in the presence of β-estradiol, (a) using Ag-NP ink as a reference, (b) Ag-SL ink as a reference, at concentrations from 1.0 to 6.0 μmol L^−1^ in PBS 0.1 mol L^−1^ (pH 6.0); Step = 9 mV s^−1^; amplitude = 80 mV s^−1^; frequency = 90 mV s^−1^; (b) Plot of analytical curve for β-estradiol; Video S1: Experimental part.
