# Supplementary material for: Development of New Simple Compositions of Silver Inks for the Preparation of Pseudo-Reference Electrodes
Source: Biosensors (Basel). 2022 Sep 16;12(9):761. doi: 10.3390/bios12090761 (PMC9497032; doi:10.3390/bios12090761)
Supplement: Supplementary file 1 [file biosensors-12-00761-s001.zip › biosensors-1899158-supplementary - for XML.pdf]

SUPPLEMENTARY MATERIAL

Development of New Simple Compositions of Silver Inks for the Preparation of Pseudo-Reference Electrodes

Jéssica R. Camargo [1], Wilson S. Fernandes-Junior [1], Déborah C. Azzi [2], Raquel G. Rocha [3], Lucas V. Faria [3], Eduardo M. Richter [3], Rodrigo A. A. Muñoz [3,*] and Bruno C. Janegitz [1,*]

[1] Department of Nature Sciences, Mathematics and Education, Federal University of São Carlos, Araras 13600-970, SP, Brazil
[2] ADB Pesquisa e Desenvolvimento, Araras 13600-140, SP, Brazil
[3] Institute of Chemistry, Federal University of Uberlândia, Uberlândia 38400-902, MG, Brazil
* Correspondence: munoz@ufu.br (R.A.A.M.), brunocj@ufscar.br (B.C.J.)

Table S1. Compositions for the production of conductive silver ink.

Ink	Composition	Percentage (%) (w/w)
1	Powder silver and nail polish	50/50
2	Powder silver and nail polish	40/60
3	Powder silver and nail polish	30/70
4	Powder silver, shellac, and polyurethane solvent.	25/25/50
5	Powder silver, shellac, and polyurethane solvent.	33/33/33
6	Powder silver and shellac	50/50
7	Powder silver and shellac	70/30
8	Powder silver and shellac	60/40

The nail polish composition is: butyl acetate, ethyl acetate, nitrocellulose, acetyl tributyl, citrate, toluene, isopropyl alcohol, tosylamide/formaldehyde resin, stearalkonium hectorite, alcohol, hydrated silica, and mineral oil. The nail polish can

also contain: CI 42090,15850, 74160, 45410, 15850, 77007, 77491, 15985, 77266, 19140, 77891, 15880, 45380, 47005,77000,77019, 60730, polyethylene terephthalate, acrylates copolymer, panthenol, and argania spinosa kernel oil. The shellac is only composed of natural resins and solvents, according to the label.

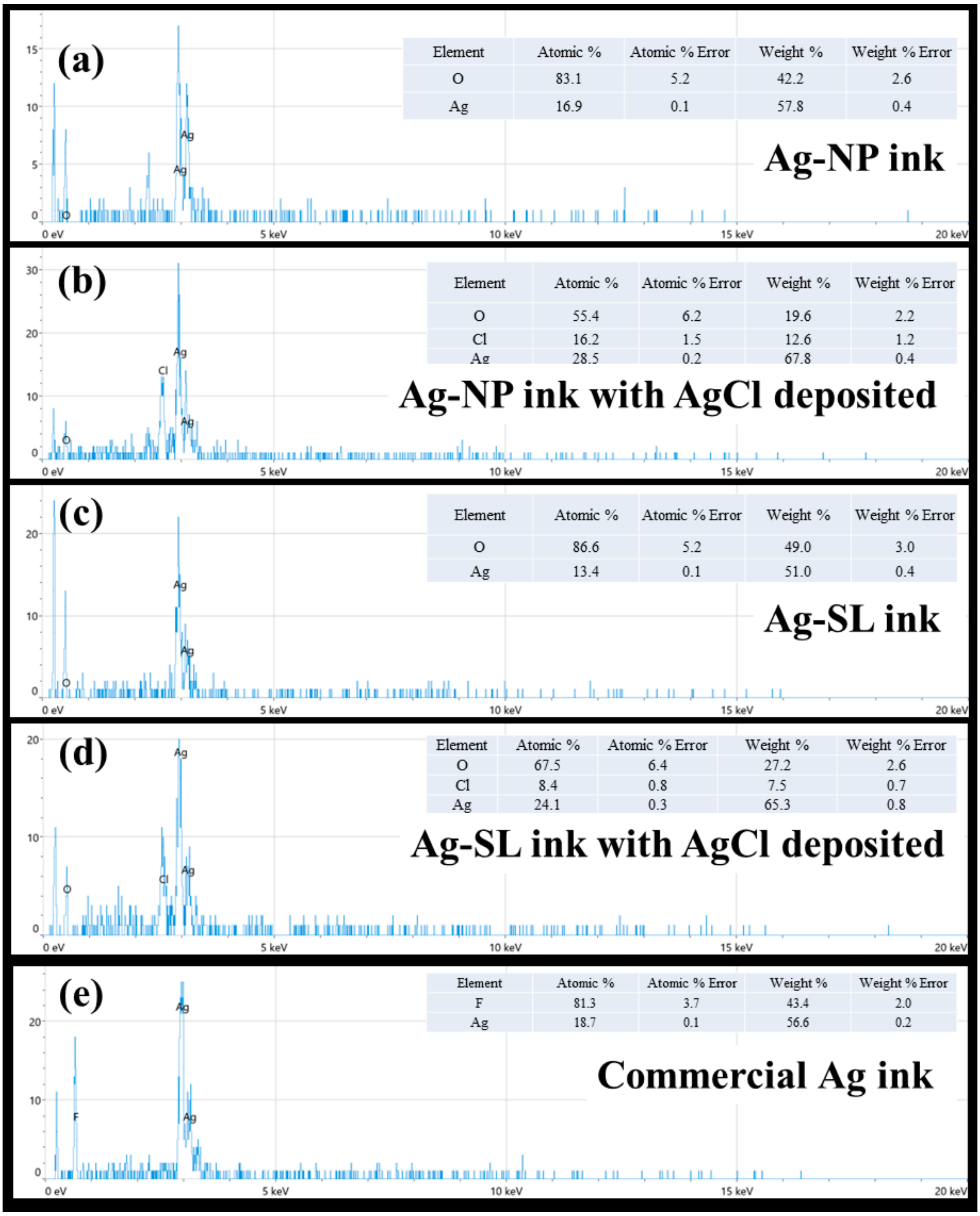

Figure S1. EDS mapping of (a) Ag-NP ink; (b) Ag-NP ink with AgCl deposited; (c) Ag-SL ink; (d) Ag-SL ink with AgCl deposited and (e) Ag commercial ink.

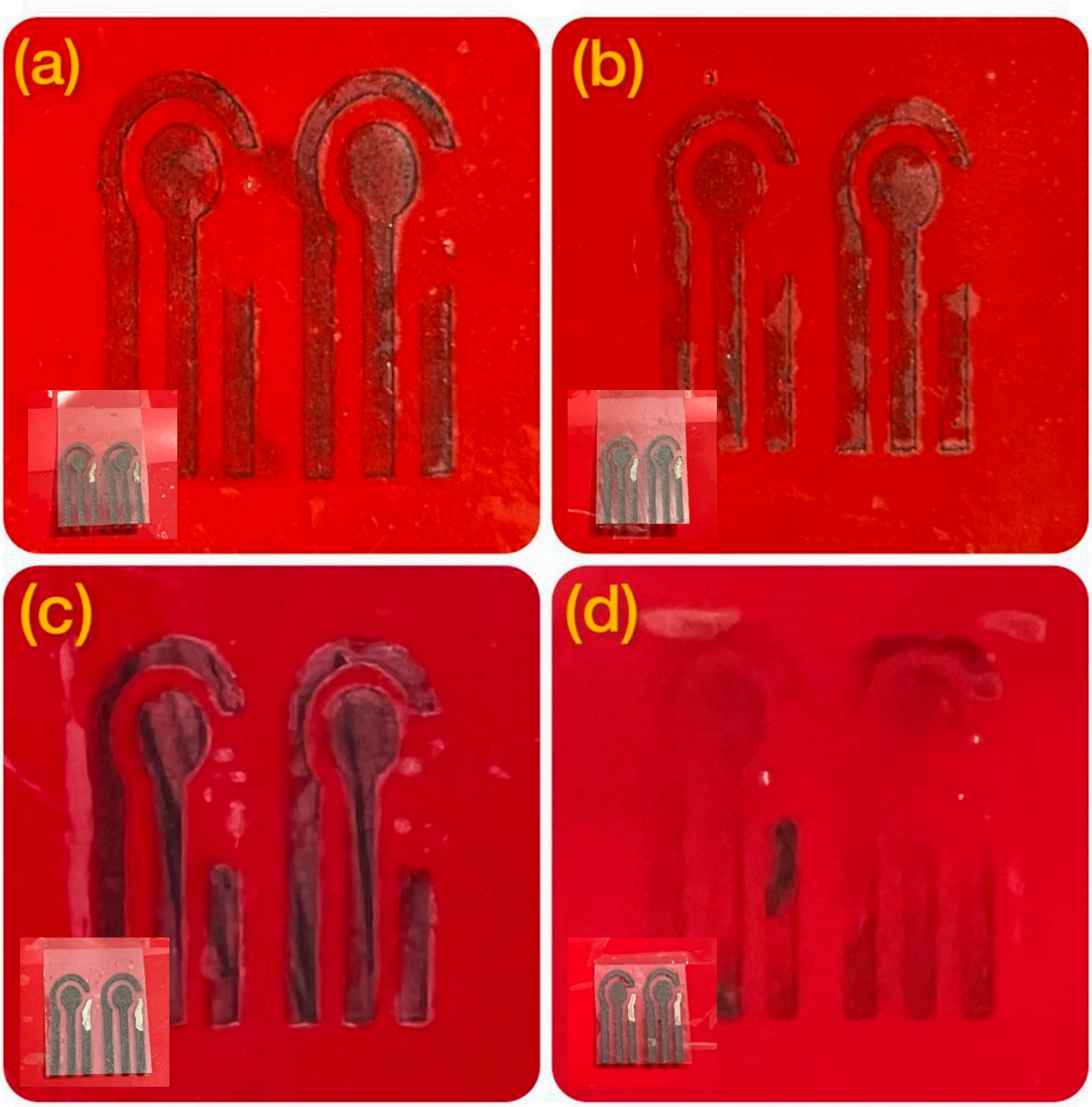

Figure S2. Pictures of Adhesive Tape Test were performed using the SPE-type system and the conductive Ag inks used over the reference electrode. Here, (a) represented the image of the adhesive removed at 45°, and (b) 90° using the carbonaceous ink of graphite and SL and the Ag-SL ink. Above, (c) and (d) represent the image obtained from the adhesive removed at 45° and 90°, respectively, using the carbonaceous ink of graphite and SL and Ag-NP ink. *Inset*: photos of SPE devices after pulling.

Table S2. Charge transfer resistance values for proposed Ag inks

System	Charge transfer resistance II (KΩ)	Circuit Description	Chi-square χ^2
3D with Ag-NP ink	572	[R([RG]Q)(RC)]	0.075035
3D with Ag-SL ink	25.3	[R([RG]Q)(RC)]	0.014301
Acetate sheets with Ag-NP ink	131	[R([RG]Q)(RC)]	0.003511
Acetate sheets with Ag-SL ink	15.7	[R([RG]Q)(RC)]	0.029912
SPE with Ag-NP ink	38.1	[R([RWG]Q)(RC)]	0.025123
SPE with Ag-SL ink	2.31	[R([RWG]Q)(RC)]	0.018496

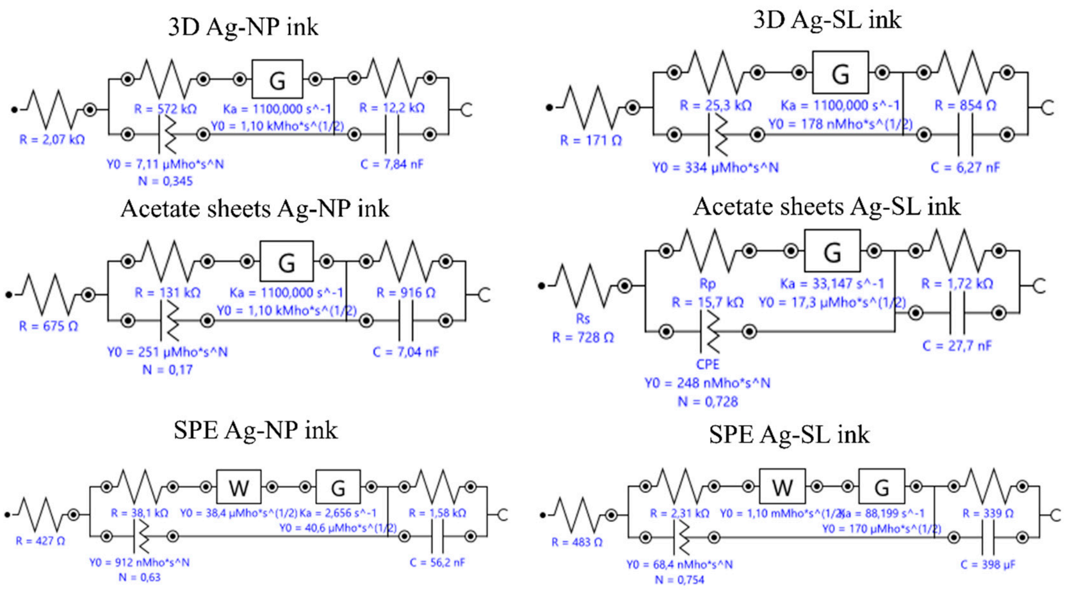

Figure S3. Equivalent circuits obtained after EIS technique for the Ag inks with systems (3D, acetate sheets, and SPE).

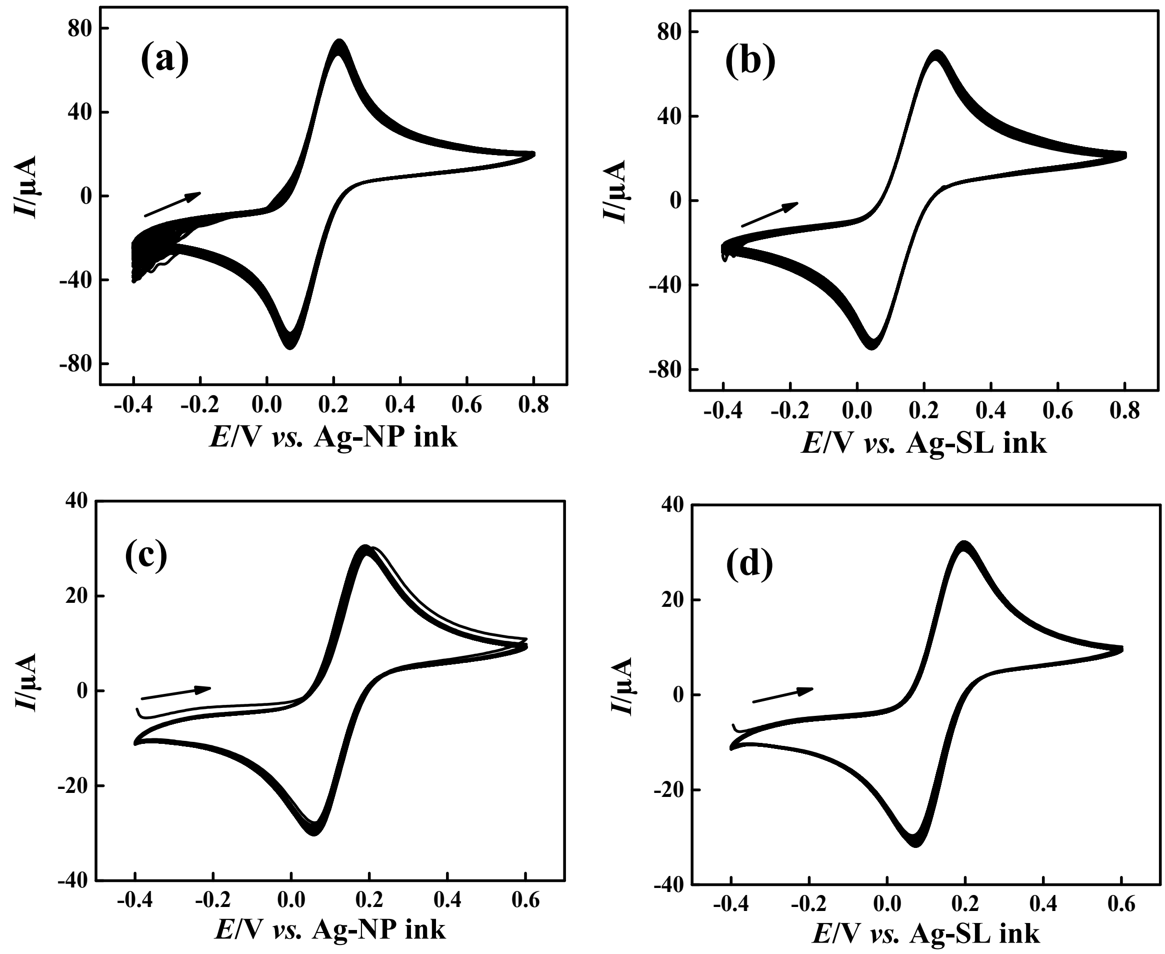

Figure S4. Successive cyclic voltametric measurements (n=100) obtained for 1.0 mmol L^{-1} ferrocenemethanol-varying reference electrode. (a) and (b) represent the working electrode: GP-SL/Ac; (c) and (d) 3D-printed CB/PLA; Scan rate: 100 mV s^{-1}

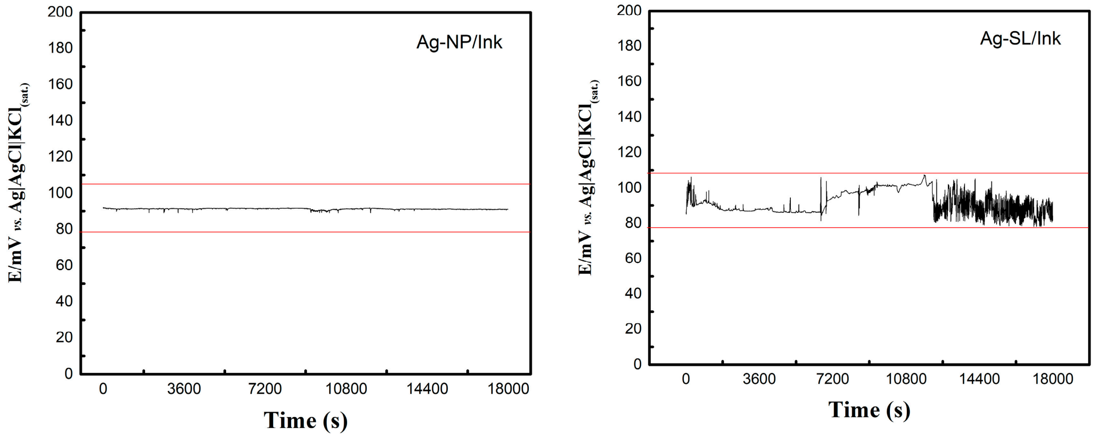

Figure S5. OCP measurements recorded for 5h in the GP-SL/AC system using Ag-NP ink and Ag-SL ink as working electrodes, Ag|AgCl|$KCl_{(sat.)}$ as the reference electrode, and 0.1 mol L^{-1} KCl solution as the supporting electrolyte.

Analytical curves were constructed for both inks using different systems, the screen-printed GP-SL/Ac and 3D-printed CB/PLA sensor. The estimated limit of detection (LOD) for β-estradiol was calculated as LOD = 3 × SD/S, where SD is the blank standard deviation and S is the sensitivity. For the GP-SL/Ac system referenced by Ag-NP ink (Figure S6a), following the equation: I (μA) = 0.07 C (β-estradiol) + 1.56 × 10^{-6}, with R^2 = 0.998, and limit of detection 1.3 × 10^{-7} mol L^{-1}. As for the system referenced by Ag-SL ink (Figure S6b), following the equation: I (μA) = 0.04 C (β-estradiol) + 2.70 × 10^{-6}, with R^2 = 0.992, and limit of detection of 9.5 × 10^{-7} mol L^{-1}.

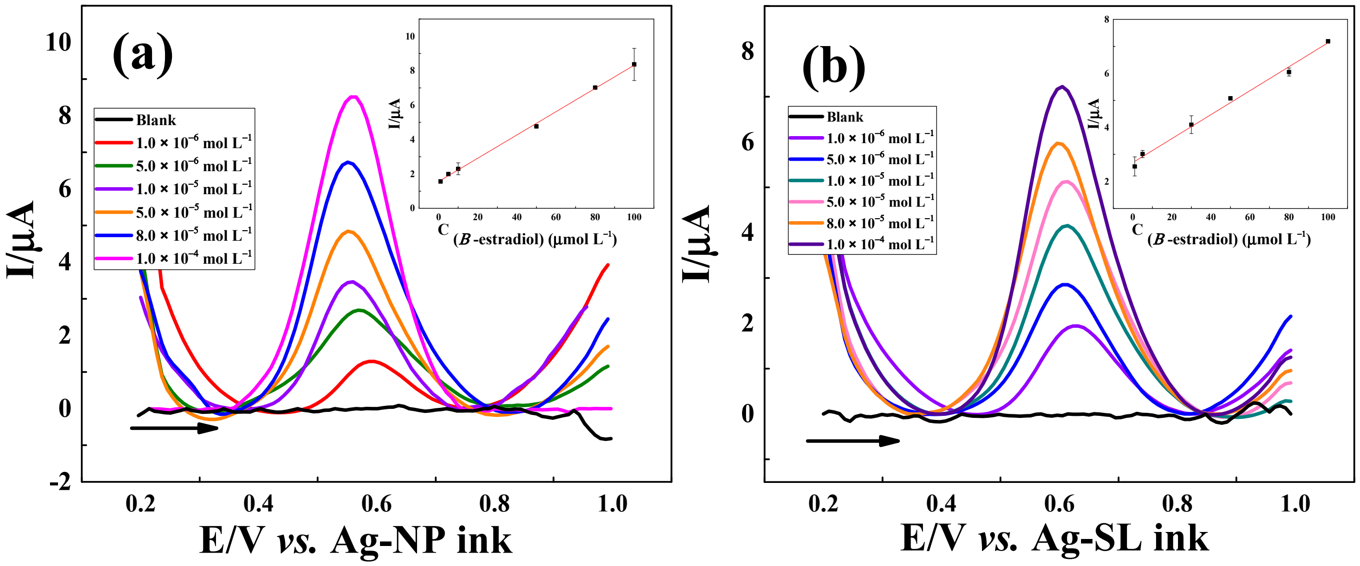

Figure S6. Square wave voltammograms in the presence of β-estradiol, (a) using Ag-NP ink as a reference and (b) Ag-SL ink as a reference at concentrations from 1.0 to 100

μmol L^{-1} in PBS 0.1 mol L^{-1} (pH 6.0); Step = 9 mV s^{-1}; amplitude = 80 mV s^{-1}; frequency = 90 mV s^{-1}; (b) plot of analytical curve for β-estradiol.

For the 3D-printed CB/PLA system referenced by Ag-NP ink (Figure S7a), following the equation: I (μA) = 0.87 C (β-estradiol) + 6.27 × 10^{-8}, with R^2 = 0.998. As for the system referenced by Ag-SL ink (Figure S7b), following the equation: I (μA) = 1.01 C (β-estradiol) – 9.23 × 10^{-8}, with R^2 = 0.999.

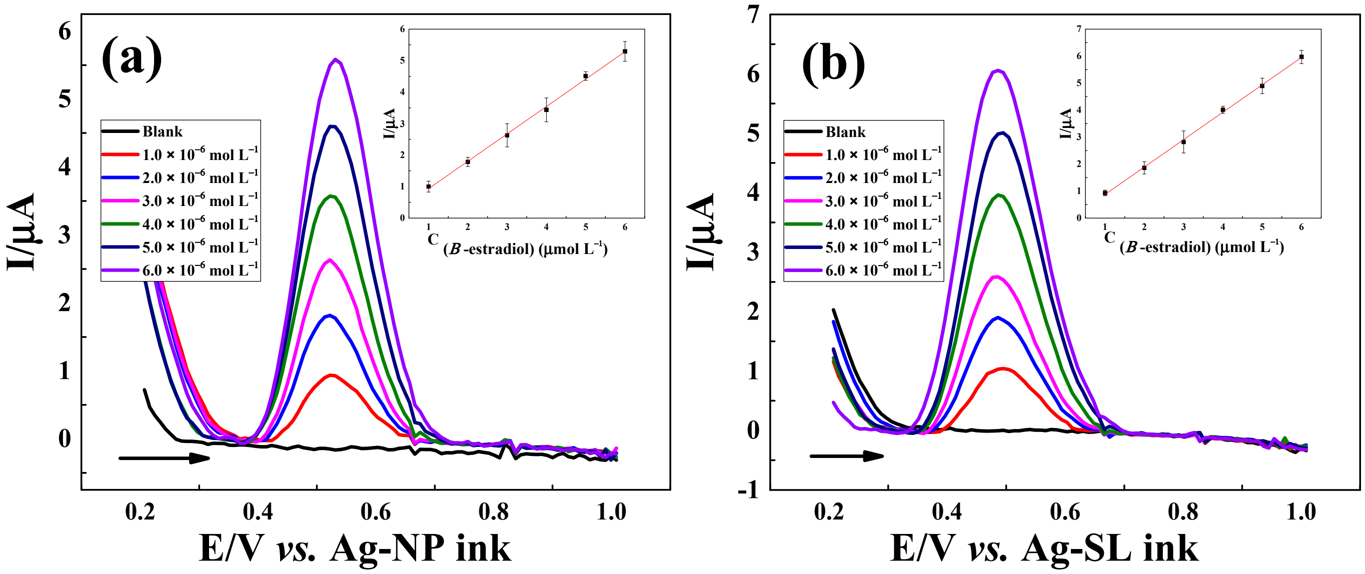

Figure S7. Square wave voltammograms in the presence of β-estradiol, (a) using Ag-NP ink as a reference and (b) Ag-SL ink as a reference, at concentrations from 1.0 to 6.0

μmol L^{-1} in PBS 0.1 mol L^{-1} (pH 6.0); Step = 9 mV s^{-1}; amplitude = 80 mV s^{-1}; frequency = 90 mV s^{-1}; (b) plot of analytical curve for β-estradiol.
